# Preliminary exploration of a quantitative assessment index for the matching performance of anatomical bone plates using computer

**DOI:** 10.1186/s13018-019-1229-3

**Published:** 2019-07-04

**Authors:** Xuhua Wu, Qingquan Xia, Ke Rong, Minfeng Gan, Gen Wen, Xiaofan Yin, Huilin Yang

**Affiliations:** 1 Department of orthopedics, The First Affiliated Hospital of Soochow University, No.899, Pinghai Road, Soochow, 215006 China; 2 Department of orthopedics, Minhang Hospital, Fudan University, No.170, Xinsong Road, Shanghai, 201199 China

**Keywords:** Anatomical bone plate, Matching performance, Quantitative index

## Abstract

**Background:**

To explore a new quantitative index to assess the matching performance of anatomical bone plates using digital technology.

**Methods:**

CT data of normal tibias of 40 adults were collected. Two brands of medial distal tibia plates were digitized. Two trained orthopedists simulated the surgical operation in Rhino 5.1 software by placing the plate curve on the medial distal tibia surface. The volume of the interstice between the plate curve and the bone surface was measured. The inverse value of this average interstice distance was used as the matching performance index (MPI). A wall thickness analysis tool was used to mark various interstice distances with varied colors.

**Results:**

The Kangli medial distal tibia plate had a MPI of 0.55 ± 0.08 by operator A and 0.55 ± 0.06 by operator B. The general care medial distal tibia plate had a MPI of 0.32 ± 0.06 by operator A and 0.31 ± 0.05 by operator B. There were significant variations in the MPI between the two types of plates by both operators (*p* < 0.001). And significant variations were observed in the MPI of general care medial distal tibia plates among various operator groups (*p* = 0.028).

**Conclusion:**

This quantitative index of matching performance is straightforward and intuitive. However, we still need a method to improve the experimental repeatability, especially when it comes to a plate with poor matching performance.

## Introduction

Numerous designs of anatomical bone plates are available for limb fractures, particularly, periarticular fractures, for matching the fracture form of the patient. For a specified thickness of the bone plate, an increase in the number of anatomical bone plates (used for fracture fixation) matching the patient’s bone, reduces the likelihood of discomfort caused by the plate. The matching performance of the bone plate can directly affect the somatosensory system and limb activity of the patient, particularly when periarticular fractures are involved. However, a bone plate is not capable of adequately matching the bone surface of all the patients due to the variations of bone structure among individuals and among the various human races. Therefore, mass-produced anatomical bone plates are not likely to be suitable for all patients. Currently, no quantifiable generic index is available for assessing the matching performance of mass-produced anatomical bone plates; moreover, detection and supervision in this regard is inadequate. Clinical use of mass-produced anatomical bone plates involves potential risks. In this study, a new quantitative assessment method and index is used to compare the matching performance of two types of anatomical bone plates; moreover, a preliminary discussion is undertaken on the feasibility of this quantitative assessment index.

## Materials and methods

### Patients

Thin-slice CT scanning data (Siemens 64-slice spiral CT, Dicom format) of normal tibias of 40 adults (20 male and 20 female cases, who suffered contralateral lower limb fracture that need to perform CT scan) were extracted from CT images obtained between April 2015 and Mar 2018. The slice thickness was 0.6 mm. The average age of the patients was 36.4 years (ranging from 25 to 52 years), tibia fractures were absent, and cases involving lower limb deformity, osteoarthritis, and tumor were excluded.

### Experimental design

**Fig. 1:**
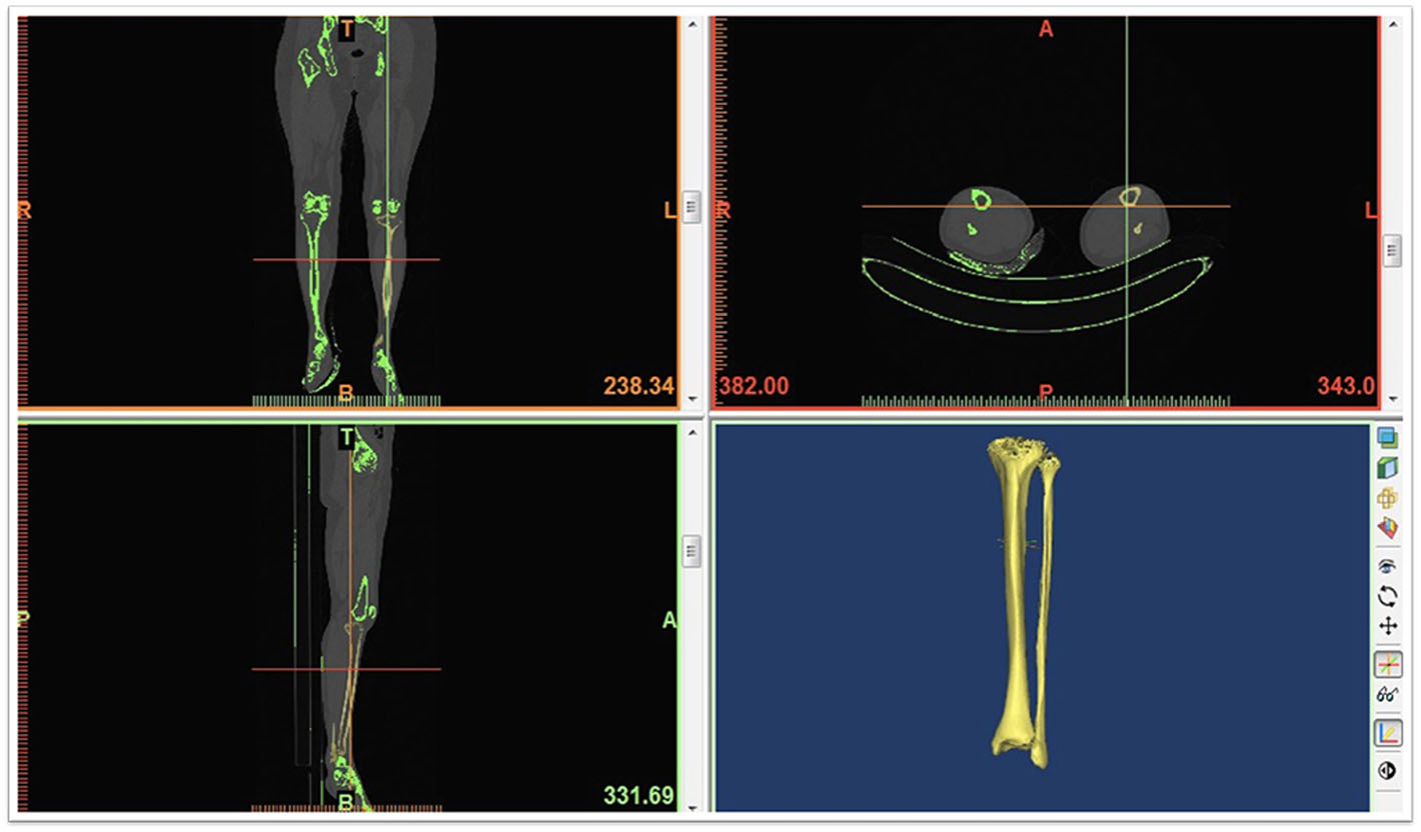
3D reconstruction of the tibia using mimics 18.0

**Fig. 2:**
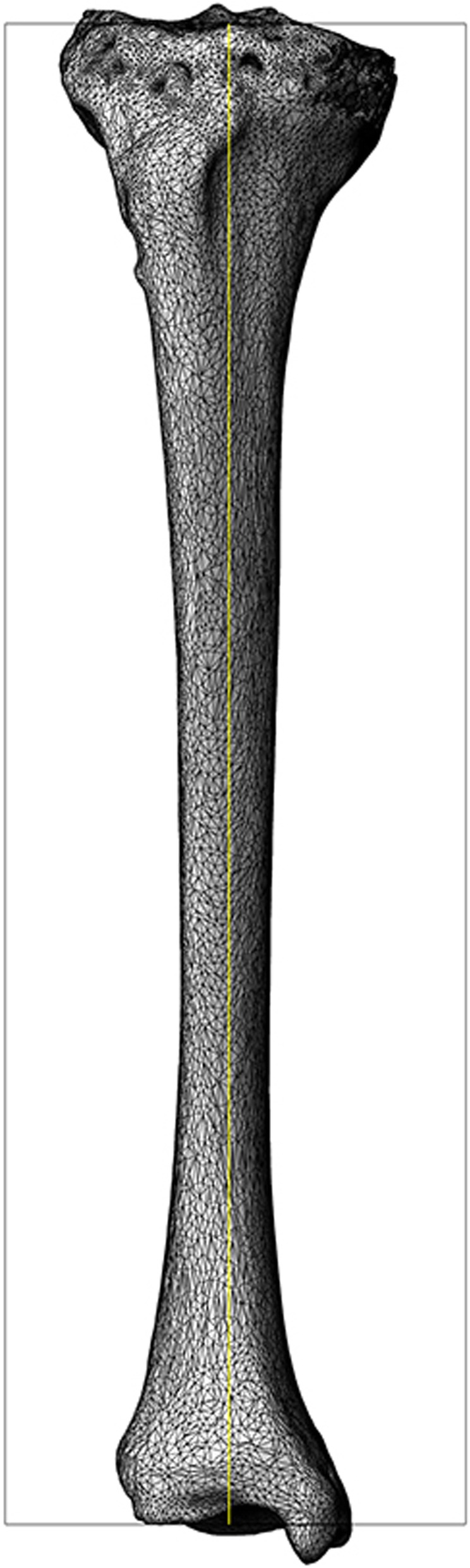
Exporting the tibia model in STL format and measuring the tibia length

The 40 cases of tibia CT data were imported into an interactive medical image control system 18.0 (MIMICS 18.0 by Materialise, Belgium). The threshold was set to 180–3071 HU, and the bone tissues were separated using the thresholding tool. The tibia was reconstructed in 3D using the Calculate 3D tool (Fig. 1). The morphology operation tool was used to fill the small voids on the surface and smoothen the surface of the bone structure. The tibia model (Fig. 2) was then exported in STL format, and the length of the tibia was measured.

**Fig. 3:**
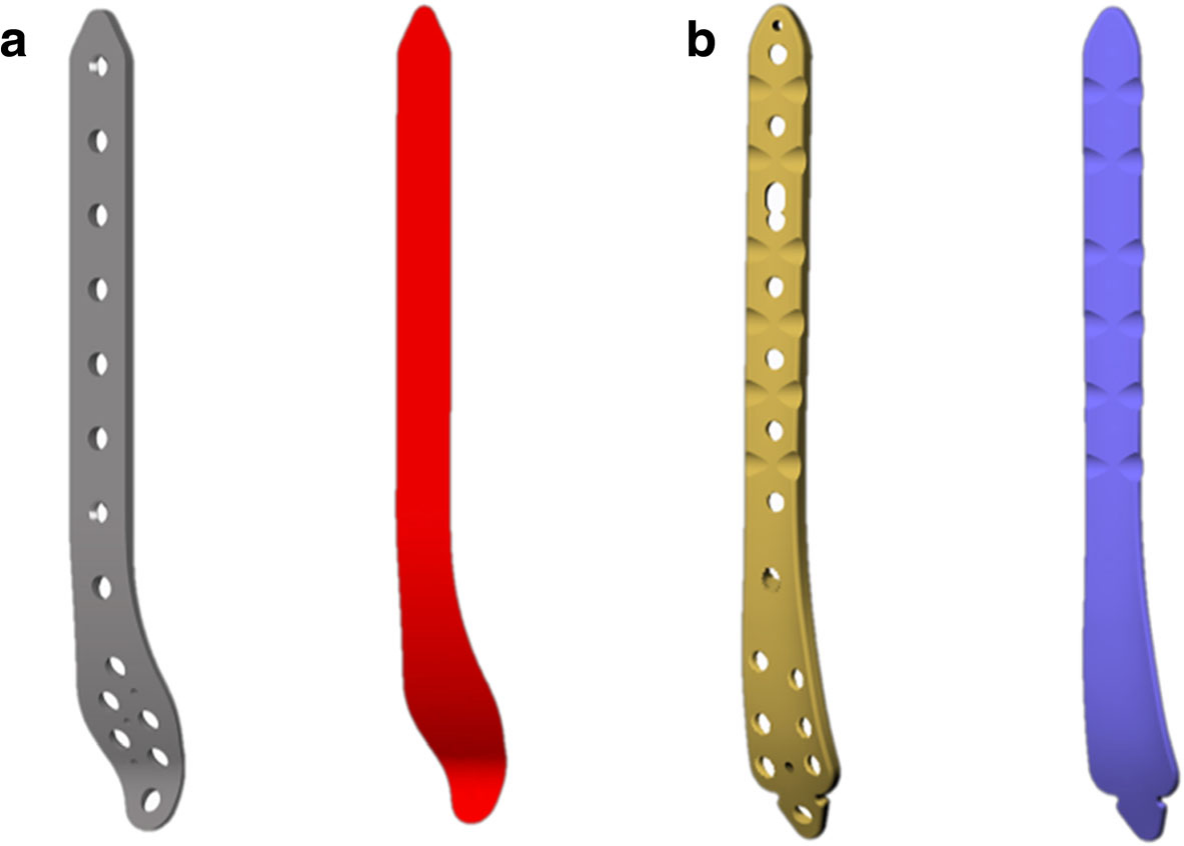
**a** Modeling the general care tibia medial 8-hole anatomical bone plate and extracting the surface on the bone side. **b** Modeling the Kangli tibia medial 8-hole anatomical bone plate and extracting the surface on the bone side

**Fig. 4:**
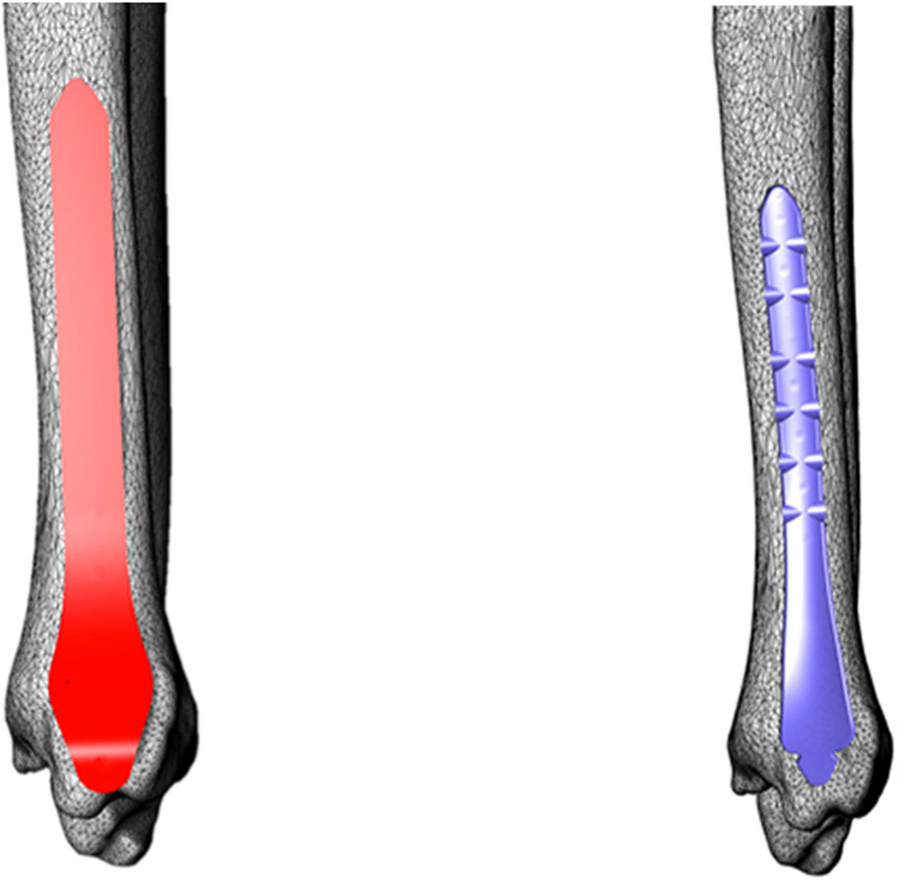
Simulating the surgical operation: placing the bone-facing curves of the two types of bone plates on the medial distal tibia in such a way that the bone plate curves are as close to the bone surface as possible

Two commercial bone plates were used for the purposes of this study. Rhino 5.1 (Robert McNeel, USA) was used to perform a proportional 3D digital modeling of the general care distal medial tibia 8-hole anatomical bone plate (general care International, Lyon, France) and the Kangli distal medial tibia 8-hole anatomical bone plate (Suzhou Kangli Orthopaedics Instrument Co., Ltd., China). The bone-facing curves were extracted and the screw holes were filled (Fig. 3). Two trained orthopedists simulated the surgical operation by placing the plate curve on the medial distal tibia surface and adjusted the position until the optimal attachment state was attained (Fig. 4). In order to make the placement of the plate as standardized as possible, three criteria must be followed as explained below:

1. The axis of the plate should be parallel to the axis of the tibia at the sagittal plane.
2. The plate should be placed as close as possible to the bone surface, and it should be made sure that there is at least one point that sticks to the bone surface.
3. The distance between the distal end of the plate and the bone surface should be no greater than 2 mm.

**Fig. 5:**
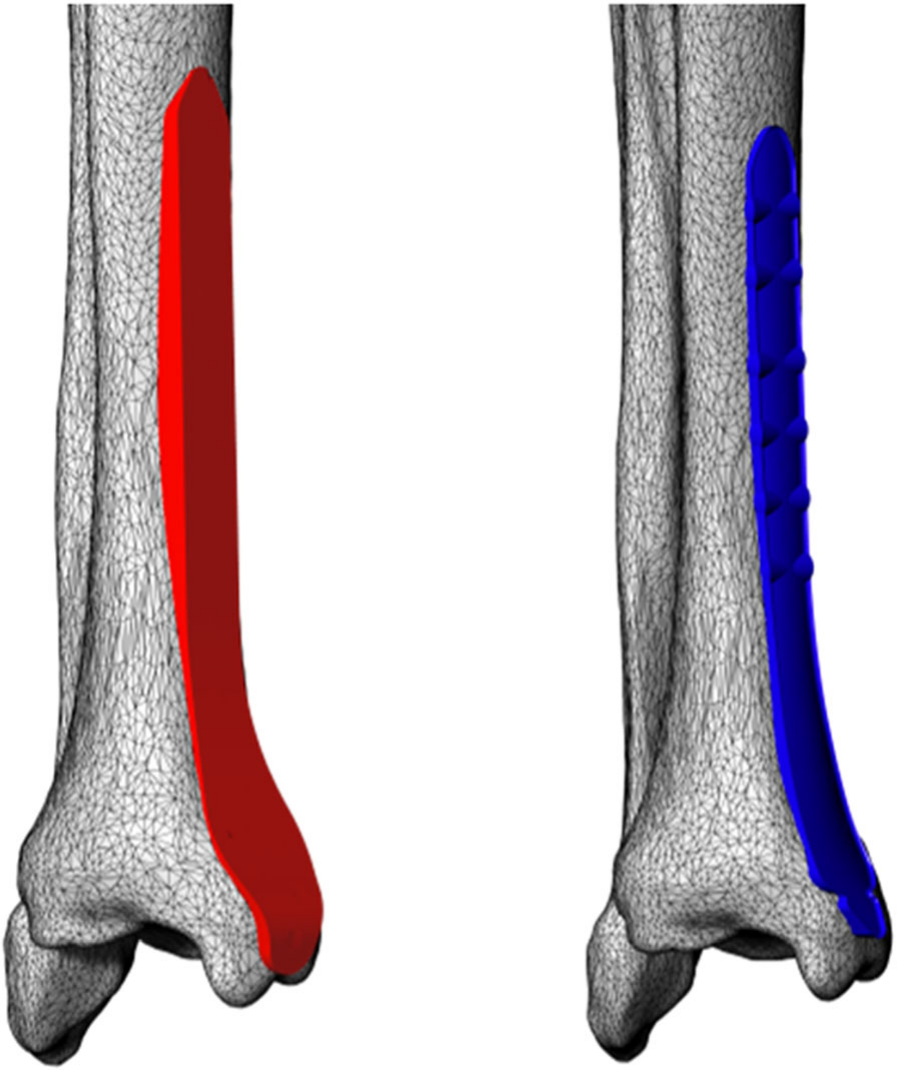
Using the surface extrusion tool to extrude the longitudinal axle vertical to the tibia in order to form a solid

**Fig. 6:**
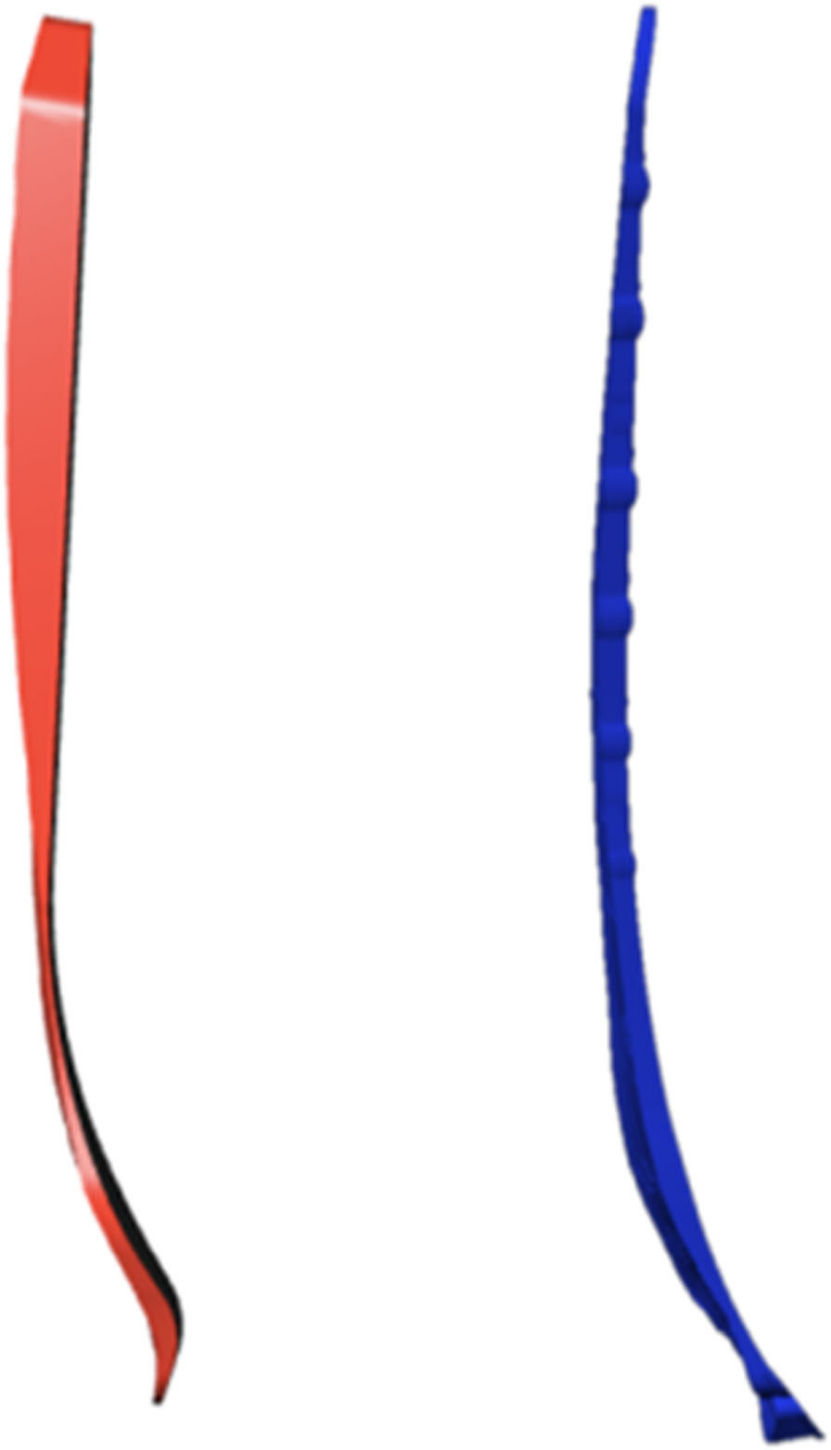
Determination of the interstice between the bone plate curve and the bone surface using Boolean calculation

The surface extrusion tool in solid tools was used to extrude the longitudinal axle and generate a solid vertical to the tibia to facilitate the overlapping of the solid and the bone (Fig. 5). It is feasible to determine the form of the interstice between the bone curve and the anatomical plate surface using a Boolean calculation (Fig. 6). The volume of the interstice was calculated by the solid analysis tool, and the average interstice distance was obtained by dividing the interstice volume by the surface area of the bone plate. The average interstice distance is inversely proportional to the matching performance of the bone plate. The smaller the interstice between the bone plate and the bone, the higher is the matching performance, and vice versa. Therefore, this study used the reciprocal of the average interstice distance as a quantitative matching performance index (MPI) in order to assess an anatomical bone plate. A higher value of this index represents higher matching performance.

The matching performance formula is as follows:

1 $$ \mathrm{Matching}\ \mathrm{performance}\ \mathrm{index}\ \left(\mathrm{MPI}\right)=\frac{1}{\mathrm{Average}\ \mathrm{interstice}\ \mathrm{distance}} = \frac{\mathrm{Surface}\ \mathrm{area}\ \mathrm{of}\ \mathrm{bone}-\mathrm{facing}\ \mathrm{plate}}{\mathrm{Interstice}\ \mathrm{volume}} $$

The interstice model was exported in STL format and then imported into 3-matic Research 9.0. The wall thickness analysis tool in analysis was opened, and the parameter was set to 3 mm. With this value as the threshold, interstice distances larger than this value were marked by the color red, and those smaller than this value were marked by the color green. Thus, the various thicknesses in the interstice model were marked with varied colors.

### Statistical analysis

The statistical analysis was performed using the SPSS 18.0 statistical software (PASW Statistics, IBM, USA). Normally distributed data were expressed as mean ± standard deviation (*x* ± *s*), and the data analysis program included two independent samples: a *t* test (e.g., gender impact) and Pearson correlation analysis (plate type impact). A value of *p* < 0.05 indicates statistical significance.

## Results

**Fig. 7:**
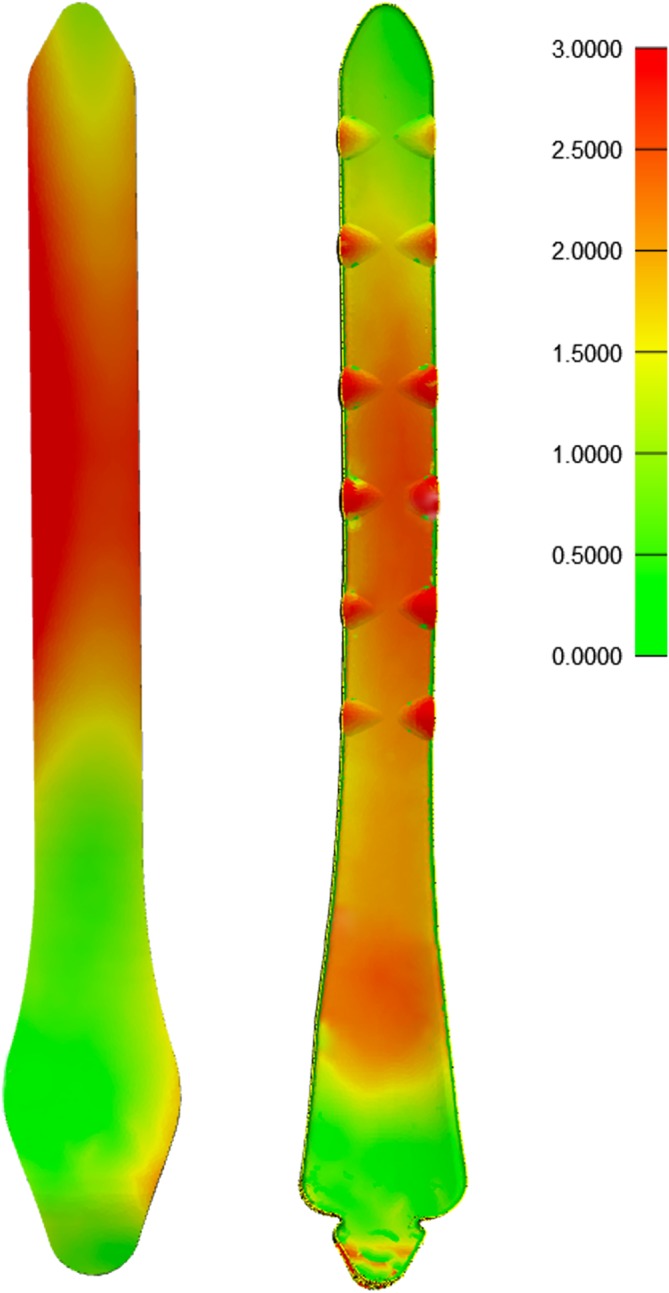
Matching performance of nephogram for anatomical bone plates. Using the wall thickness analysis tool in 3-matic Research 9.0 to mark parts with various thicknesses in the interstice model with varied colors—the smaller the interstice, the greener the color; the larger the interstice, the redder the color

For the Kangli 8-hole medial distal tibia plate, the surface area of the bone-facing curve was 2388 mm^2^, the interstice volume was 4417.8 ± 620.9 mm^3^ by operator A and 4393.9 ± 477.6 mm^3^ by operator B, the MPI was 0.55 ± 0.08 by operator A and 0.55 ± 0.06 by operator B. For the general care 8-hole medial distal tibia plate, the surface area of the bone-facing curve was 2505 mm^2^, the interstice volume was 8041.1 ± 1553.1 mm^3^ by operator A and 8241.5 ± 1252.5 mm^3^ by operator B, and the MPI was 0.32 ± 0.06 by operator A and 0.31 ± 0.05 by operator B (Table 1). In this study, there were significant variations in the MPI between the two types of plates by both operators (by operator A, *p* < 0.001; by operator B, *p* < 0.001). No significant variation was observed in the MPI of Kangli medial distal plate among various operator groups (for Kangli, *p* = 0.664). However, significant variations were observed in the MPI of general care medial distal tibia plates among various operator groups (for general care, *p* = 0.028) (Table 2). When using the quantitative index to compare the two types of plates, it was discovered that the Kangli medial distal tibia plate exhibited higher matching performance while a large red area with a relatively fixed position was observed for the general care medial distal tibia plate (Fig. 7). A significant further enhancement is feasible.

**Table 1:** Matching data and analysis for the two types of tibia distal anatomical bone plates by two operators

Sample	Gender	Tibia length (mm)	Kangli (MPI)		General care (MPI)	
			Operator A	Operator B	Operator A	Operator B
1	Male	353.00	0.53	0.56	0.29	0.31
2	Male	355.00	0.63	0.59	0.21	0.22
3	Male	357.00	0.50	0.53	0.34	0.30
4	Male	358.00	0.77	0.67	0.50	0.45
5	Male	361.00	0.63	0.59	0.43	0.42
6	Male	361.00	0.59	0.67	0.40	0.37
7	Male	363.00	0.56	0.63	0.37	0.33
8	Male	365.00	0.48	0.50	0.33	0.29
9	Male	356.00	0.71	0.67	0.28	0.25
10	Male	368.00	0.67	0.59	0.34	0.31
11	Male	366.00	0.56	0.56	0.33	0.37
12	Male	359.00	0.47	0.50	0.37	0.34
13	Male	371.00	0.63	0.63	0.34	0.31
14	Male	370.00	0.67	0.63	0.30	0.29
15	Male	360.00	0.59	0.59	0.26	0.27
16	Male	357.00	0.53	0.50	0.30	0.29
17	Male	362.00	0.48	0.50	0.24	0.26
18	Male	366.00	0.50	0.53	0.26	0.28
19	Male	358.00	0.56	0.56	0.21	0.24
20	Male	355.00	0.45	0.48	0.32	0.29
21	Female	337.00	0.59	0.56	0.36	0.32
22	Female	338.00	0.42	0.45	0.30	0.29
23	Female	338.00	0.43	0.45	0.29	0.31
24	Female	339.00	0.53	0.50	0.34	0.30
25	Female	339.00	0.50	0.50	0.30	0.33
26	Female	340.00	0.48	0.48	0.31	0.32
27	Female	340.00	0.53	0.56	0.38	0.36
28	Female	341.00	0.45	0.48	0.34	0.31
29	Female	341.00	0.67	0.63	0.32	0.34
30	Female	342.00	0.59	0.59	0.37	0.31
31	Female	345.00	0.56	0.56	0.30	0.32
32	Female	350.00	0.53	0.56	0.29	0.29
33	Female	333.00	0.59	0.56	0.30	0.29
34	Female	332.00	0.48	0.50	0.24	0.26
35	Female	346.00	0.50	0.50	0.36	0.29
35	Female	352.00	0.53	0.56	0.26	0.23
37	Female	337.00	0.59	0.56	0.24	0.26
38	Female	344.00	0.45	0.48	0.45	0.42
39	Female	350.00	0.71	0.63	0.34	0.31
40	Female	343.00	0.56	0.50	0.32	0.36
Average value		351.2 ± 11.25	0.55 ± 0.08	0.55 ± 0.06	0.32 ± 0.06	0.31 ± 0.05

**Table 2:** Significant difference observed in the tibia matching performance between different bone plates in an identical operator group

MPI				
Group	Kangli	General care	*T* value	*p* value
Operator A	0.55 ± 0.08	0.32 ± 0.06	15.863	< 0.001
Operator B	0.55 ± 0.06	0.31 ± 0.05	20.906	< 0.001
*T* value	0.438	2.283		
*p* value	0.664	0.028

## Discussion

Varying degrees of racial, gender, and individual variations exist in the skeletal morphology of the human body [1–9]; however, clinical customized 3D printed plates are not currently popular due to time and production costs. Currently, the most common anatomical bone plate for clinical use exhibits a high price–performance ratio. Moreover, a variety of anatomical bone plates are available for clinical use [10–14] and the products are becoming increasingly mature; numerous companies have claimed superiority of the matching performance of their respective anatomical bone plates. Nonetheless, a few studies on the matching performance of anatomical bone plates indicate that mismatch problems continue to exist in certain commonly used clinical anatomical bone plates [15, 16].

A standardized matching performance test, which is essential before putting mass-produced anatomical bone plates into clinical use, is unavailable. Moreover, it is challenging for the clinicians to provide a personalized evaluation of the plate matching performance for the patient before the operation. This poses a potential risk in its clinical use [17, 18]. According to existing literature, a majority of studies on the matching performance of anatomical bone plates started by measuring the bones of corpses [15, 16] or the CT images of living individuals [19]. When the measurement is conducted based on certain local surface characteristics of particular bone plates and bones, it is not feasible to quantitatively assess the comprehensive matching performance of the anatomical bone plate from a global perspective. Furthermore, because there is no generic quantitative index, it is not feasible to quantitatively compare the matching performances of various types of anatomical bone plates.

With the development of the digital orthopedic platform, the quantitative assessment of the matching performance of anatomical bone plates using the computer has been made feasible [20–22]. In this study, a 3D reconstruction of the bones of a normal population was performed by CT while the surgical operation was simulated to calculate the interstice volume between the plate and the bone. The reciprocal of the average distance between the bone-facing plate curve and the corresponding bone surface is used as a quantitative index of the matching performance. It is considered that the average interstice distance is inversely proportional to the matching performance; a higher index value represents higher matching performance. However, this index is not affected by the surface area of the bone plate; therefore, various anatomical bone plates can be compared in parallel. With the wall thickness analysis tool of the 3-magic Research kit in Mimics 18.0, areas with large interstice distance can be visually identified. For areas with high matching requirements, such as periarticular parts, special attention must be accorded. The process of placing the plate in the software requires the services of an orthopedist, while the plate is to be constantly adjusted until the optimal position is attained. Owing to the reliance on the experience of an orthopedist for the placement of the bone plate, the placement is significantly affected by subjective judgment and operation. With the future development of computer technology, this operation could be simulated by computer artificial intelligence: by calculating the curve of the anatomical bone plate, the plate will be automatically placed at the position most suitable for the target bone surface. The MPI of the bone plate and the bone surface will be calculated; thus, the subjective factors of the operator will be eliminated in order to achieve higher repeatability.

The method and index used in this study are straightforward and intuitive and are highly suitable for the quantitative assessment of matching the performance of anatomical bone plates. It is also suitable for use in the selection and comparison of anatomical plates before the clinical operation. The proper use of the computer in a virtual environment or that of a computer control mechanism in an actual environment to place the anatomical plate accurately in the optimum position remains a technical challenge that urgently requires a solution. We still need a method to improve the experimental repeatability, especially when it comes to a plate with poor matching performance.

## Data Availability

The datasets used and/or analyzed during the current study are available from the corresponding author on reasonable request.

## Abbreviations

CT: Computed tomography
MPI: Matching performance index
